# FHIR-Ontop-OMOP: Building clinical knowledge graphs in FHIR RDF with the OMOP Common data Model

**DOI:** 10.1016/j.jbi.2022.104201

**Published:** 2022-09-09

**Authors:** Guohui Xiao, Emily Pfaff, Eric Prud’hommeaux, David Booth, Deepak K. Sharma, Nan Huo, Yue Yu, Nansu Zong, Kathryn J. Ruddy, Christopher G. Chute, Guoqian Jiang

**Affiliations:** a University of Bergen, Norway; b University of Oslo, Norway; c Ontopic S.r.l., Italy; d University of North Carolina, Chapel Hill, NC, USA; e Janeiro Digital, Boston, MA, USA; f Yosemite Project, Somerville, MA, USA; g Mayo Clinic, Rochester, MN, USA; h Johns Hopkins University, Baltimore, MD, USA

**Keywords:** Fast Healthcare Interoperability Resources (FHIR), Data Standards, Semantic Web, Clinical Knowledge Graphs, Virtual Knowledge Graphs, Shape Expressions (ShEx)

## Abstract

**Background:**

Knowledge graphs (KGs) play a key role to enable explainable artificial intelligence (AI) applications in healthcare. Constructing clinical knowledge graphs (CKGs) against heterogeneous electronic health records (EHRs) has been desired by the research and healthcare AI communities. From the standardization perspective, community-based standards such as the Fast Healthcare Interoperability Resources (FHIR) and the Observational Medical Outcomes Partnership (OMOP) Common Data Model (CDM) are increasingly used to represent and standardize EHR data for clinical data analytics, however, the potential of such a standard on building CKG has not been well investigated.

**Objective:**

To develop and evaluate methods and tools that expose the OMOP CDM-based clinical data repositories into virtual clinical KGs that are compliant with FHIR Resource Description Framework (RDF) specification.

**Methods:**

We developed a system called FHIR-Ontop-OMOP to generate virtual clinical KGs from the OMOP relational databases. We leveraged an OMOP CDM-based Medical Information Mart for Intensive Care (MIMIC-III) data repository to evaluate the FHIR-Ontop-OMOP system in terms of the faithfulness of data transformation and the conformance of the generated CKGs to the FHIR RDF specification.

**Results:**

A beta version of the system has been released. A total of more than 100 data element mappings from 11 OMOP CDM clinical data, health system and vocabulary tables were implemented in the system, covering 11 FHIR resources. The generated virtual CKG from MIMIC-III contains 46,520 instances of FHIR Patient, 716,595 instances of Condition, 1,063,525 instances of Procedure, 24,934,751 instances of MedicationStatement, 365,181,104 instances of Observations, and 4,779,672 instances of CodeableConcept. Patient counts identified by five pairs of SQL (over the MIMIC database) and SPARQL (over the virtual CKG) queries were identical, ensuring the faithfulness of the data transformation. Generated CKG in RDF triples for 100 patients were fully conformant with the FHIR RDF specification.

**Conclusion:**

The FHIR-Ontop-OMOP system can expose OMOP database as a FHIR-compliant RDF graph. It provides a meaningful use case demonstrating the potentials that can be enabled by the interoperability between FHIR and OMOP CDM. Generated clinical KGs in FHIR RDF provide a semantic foundation to enable explainable AI applications in healthcare.

## 1. Introduction

Artificial intelligence (AI) offers significant potential for improving healthcare. As healthcare is a safety–critical industry, there is a growing demand for AI applications that are not only well-performing, but trustworthy, transparent, interpretable, and explainable [1]. Three technologies Semantic Web, knowledge graphs, and data standards play an important role for enabling explainable AI in healthcare. Tim Berners-Lee envisioned the Semantic Web as a killer application to unify content being published online, through 1) tagging content with unique identifiers or Uniform Resource Identifiers (URIs); 2) representing the content utilizing well-formed definitions from taxonomies and ontologies; 3) borrowing from the knowledge representation world to utilize structuring mechanisms for data [2]. The Resource Description Framework (RDF) and ontologies are two enablers for the Semantic web, in which RDF serves as the lingua franca for exchanging machine-processable information, and ontologies provide the formal definition that allows both machines and human beings to understand the intent of the information. The strengths of the Semantic Web to explainable AI include: 1) enabling data sharing and achieving a semantic understanding of digital content; 2) tacking the provenance aspect (e.g., using Provenance Ontology in the semantic representation) and trace aspect (e.g., supporting reasoning mechanism to generate trace) of explainability; and 3) making textual content more accessible in knowledge graphs via semantic representations [3–6].

A knowledge graph (KG) is a collection of facts where entities (nodes) are connected with typed relationships. The scope of the knowledge captured by a KG may involve generic domains (e.g., Wikidata, DBPedia) or a specific domain (e.g., Bio2RDF and UMLS). The inherent inter-connectivity of KGs enables the use of network analysis and machine learning techniques to unveil hidden patterns and infer new knowledge [7]. Furthermore, studies have shown that KGs are computationally efficient and scale to very large sizes [8].

KGs play a key role to enable explainable AI applications as KGs have great potential in the design of novel neural network architectures that natively encode explanations, e.g., by adding logic representation layers in artificial neural networks, or encoding the semantics of inputs, outputs and their properties. In the context of healthcare, KGs have been already used in integrating clinical data with proteomics data for clinical decision making support and learning a KG from electronic health records (EHRs) for building medicine and self-diagnostic symptom checkers, and other different scenarios, such as treatment recommendations, medicine recommendations, drug-to-drug similarity measurements, and COVID-19 research [9–12]. Many of these applications are performed through a link prediction process by learning embeddings (i.e., low-dimensional representations) of medical entities and relations from EHRs

Data standards are another enabling technology for explainable AI in healthcare as new AI systems require large datasets to improve their accuracy and predictive capabilities, and the heterogeneity of clinical research data hinders data integration and data sharing in a consistent and comparable manner. In recent years, community-based standards such as the HL7 Fast Healthcare Interoperability Resources (FHIR) [13] and the Observational Health Data Sciences and Informatics (OHDSI) Observational Medical Outcomes Partnership (OMOP) Common Data Model (CDM) [14] are increasingly used to represent and standardize EHR and clinical research data for clinical data analytics. FHIR is rapidly emerging as a next generation standards framework for facilitating health care and EHR-based data exchange. In particular, Mayo Clinic has been collaborating with the FHIR and W3C HCLS community to develop the FHIR RDF representation specification and associated transformation and validation tools [15–17]. FHIR RDF has become one of the three standardized data formats in the FHIR specification and provides a standard machine-processable semantic foundation for clinical data to be linked with other data using ontologies.

The combination of FHIR, KGs and the Semantic Web enables a new paradigm to build explainable AI applications in healthcare. A few of such FHIR-based applications are emerging, including 1) a KG generation tool known as NLP2FHIR developed for standardizing and integrating unstructured and structured EHR data in FHIR [18]; 2) a FHIR-based EHR phenotyping framework using machine learning and deep learning techniques developed for effective data integration and accurate phenotyping [19]; and 3) FHIR RDF data is used to build AI algorithms to predict primary cancers, showing accurate prediction of cancer types can be achieved with existing EHR data and genetic report data [20].

However, existing clinical data are mostly stored in relational data sources. To facilitate standards-based semantic data integration, sharing and discovery in broader scientific research communities, there is a strong need to provide the FHIR-based data access and query services over such databases. In this study, we close this gap by developing the FHIR-Ontop-OMOP system, which can expose any OMOP database as a virtual Clinical KG compliant with FHIR RDF. We evaluate the faithfulness of the system by comparing patient counts identified by five pairs of SQL (over the MIMIC database) and SPARQL (over the virtual CKG) queries. We also materialize a CKG in RDF triples for 100 patients, and have validated its full conformance with the FHIR RDF specification.

## 2. Materials and methods

### 2.1. Materials

#### 2.1.1. FHIR Model Ontology and FHIR Shape Expressions

**Fig. 1.**
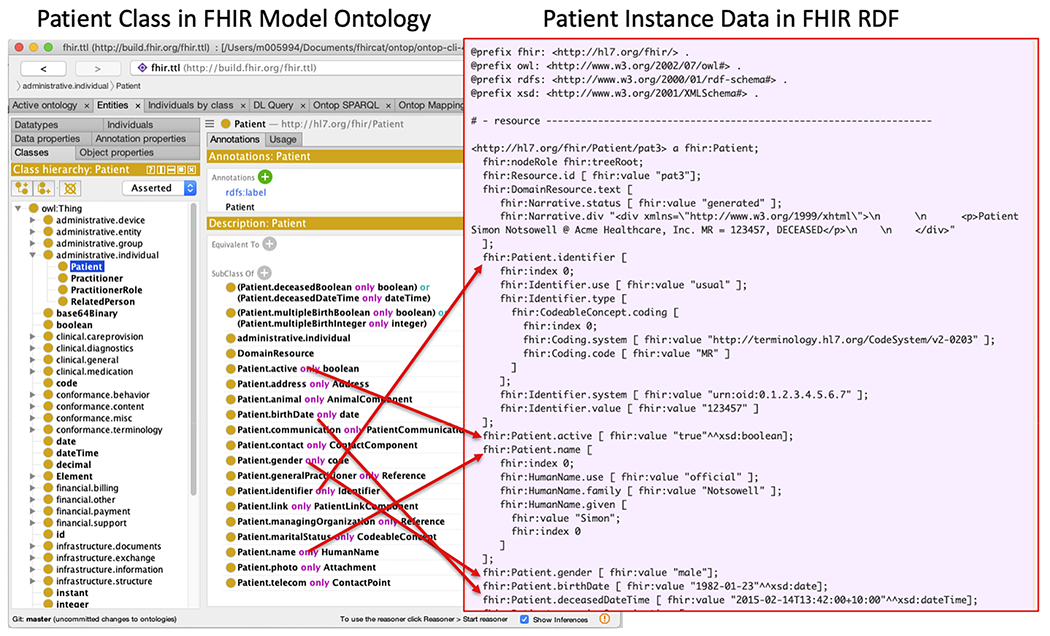
A screenshot illustrating the Patient Class definition in FHIR Model Ontology and its corresponding instance data in FHIR RDF.

One of the FHIR RDF specification efforts is to produce the FHIR *StructureDefinition* resource in the OWL ontology language, known as the “FHIR Model Ontology” [21]. The *StructureDefinition* resource is the metamodel for FHIR resource definitions, meaning that a FHIR resource such as *Patient* is formally defined using an instance of *StructureDefinition* that declares elements like “Patient.name” and “*Patient.birthDate*” and associated metadata and constraints (e.g., datatype and cardinality) [22]. The FHIR Model Ontology formally enumerates the classes, predicates, domains, ranges and specific datatypes that are used in describing the FHIR instance data in RDF. Fig. 1 shows the Patient Class definition in FHIR Model Ontology and its corresponding instance data in FHIR RDF.

**Fig. 2.**
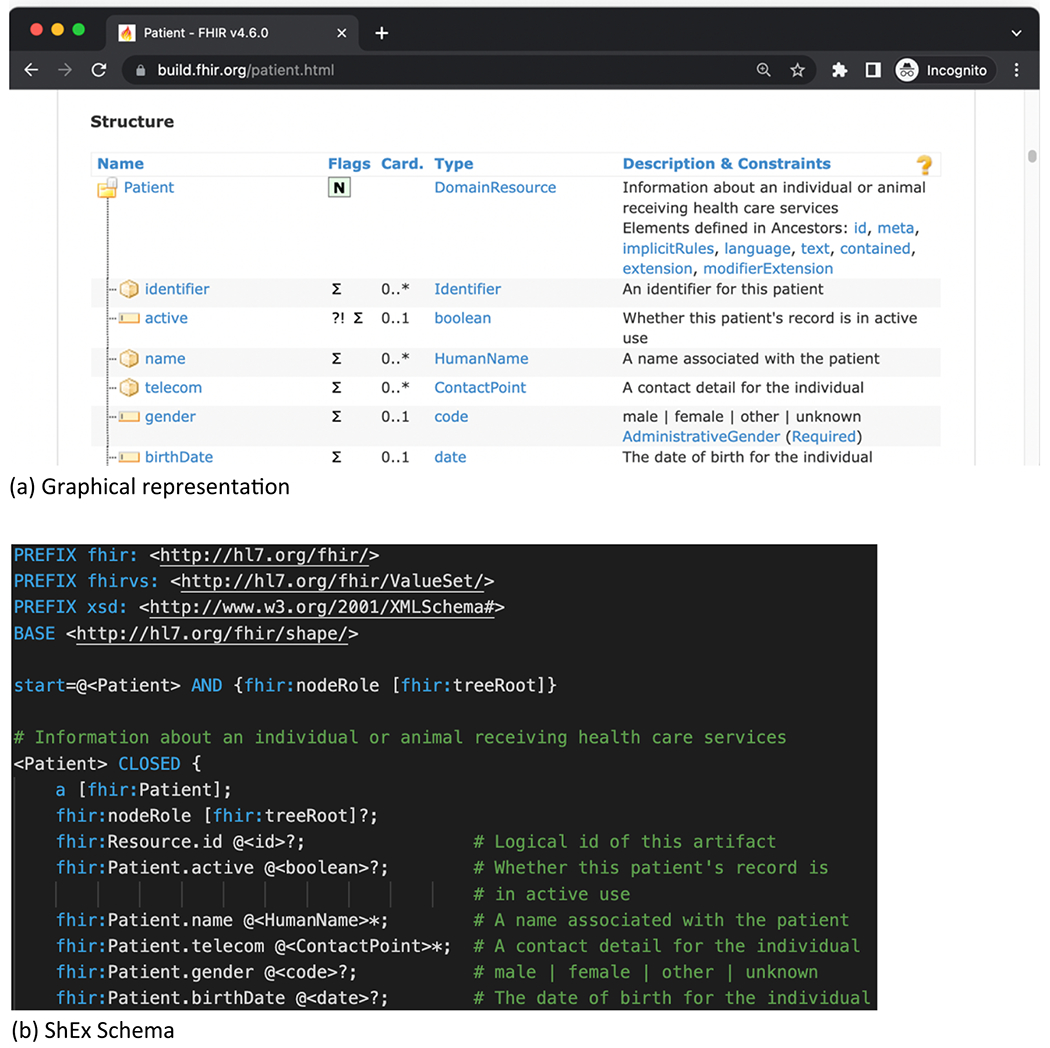
FHIR ShEx Schema of Patient.

Moreover, the FHIR definitions in the Shape Expressions Language (ShEx) can be used to test FHIR RDF graphs for conformance [15]. For example, Fig. 2 shows a graphical representation of FHIR Patient resource (Fig. 2a) and its corresponding ShEx schema (Fig. 2b). One can validate that indeed the RDF instance in Fig. 1 is compliant with this ShEx expression.

#### 2.1.2. The Ontop toolkit for virtual knowledge graphs

The Virtual Knowledge Graph (VKG) technology [23], also known as Ontology Based Data Access (OBDA) technology [24], is regarded as a key ingredient for the new generation of information systems, especially for Semantic Web applications that involve large amounts of data. The VKG approach avoids materializing triples and the query answering service is implemented through the query rewriting technique with extensive optimizations. In this approach, for a (source) database schema and a (target) ontology, a set of mappings declares how to populate the classes and the properties in the ontology. The Ontology, Mappings, and database schema together are called a VKG specification.

Ontop is the state-of-the-art open-source VKG system, which is compliant with all relevant W3C recommendations (including SPARQL 1.1 queries, R2RML mappings, and OWL2QL and RDFS ontologies), and supports for all major relational databases [25–27]. The Ontop toolkits include the Protégé Ontop Plugins to develop VKG specification [28]. Once the VKG specification is developed, we can set up a SPARQL endpoint using the command line interface of Ontop, so that end users can use standard SPARQL tools to interact with the endpoint without knowing whether the endpoint is virtual or not. There is also a dockerized Ontop endpoint to facilitate the deployment of the system.

#### 2.1.3. OMOP CDM and datasets

OMOP CDM is an open community data standard, designed to standardize the structure and content of observational data and to enable efficient analyses that can produce reliable evidence [14]. The latest CDM v5.4 is defined as a collection of standardized relational table schemas in six categories: clinical data (e.g., Person, Condition_occurrence, Drug_exposure), health system (e.g., Care_site), vocabularies (e.g., Concept, Vocabulary), health economics, derived elements, and metadata.

We used an OMOP CDM-based MIMIC-III dataset for the evaluation of the system. MIMIC-III (Medical Information Mart for Intensive Care) is a freely accessible critical care database [29]. Data includes vital signs, medications, laboratory measurements, observations and notes charted by care providers, fluid balance, procedure codes, diagnostic codes, imaging reports, hospital length of stay, survival data, and more. We used an open-source MIMIC-OMOP ETL tool to convert the MIMIC III dataset in the OMOP CDM [30].

## 3. Methods

### 3.1. System architecture

**Fig. 3.**
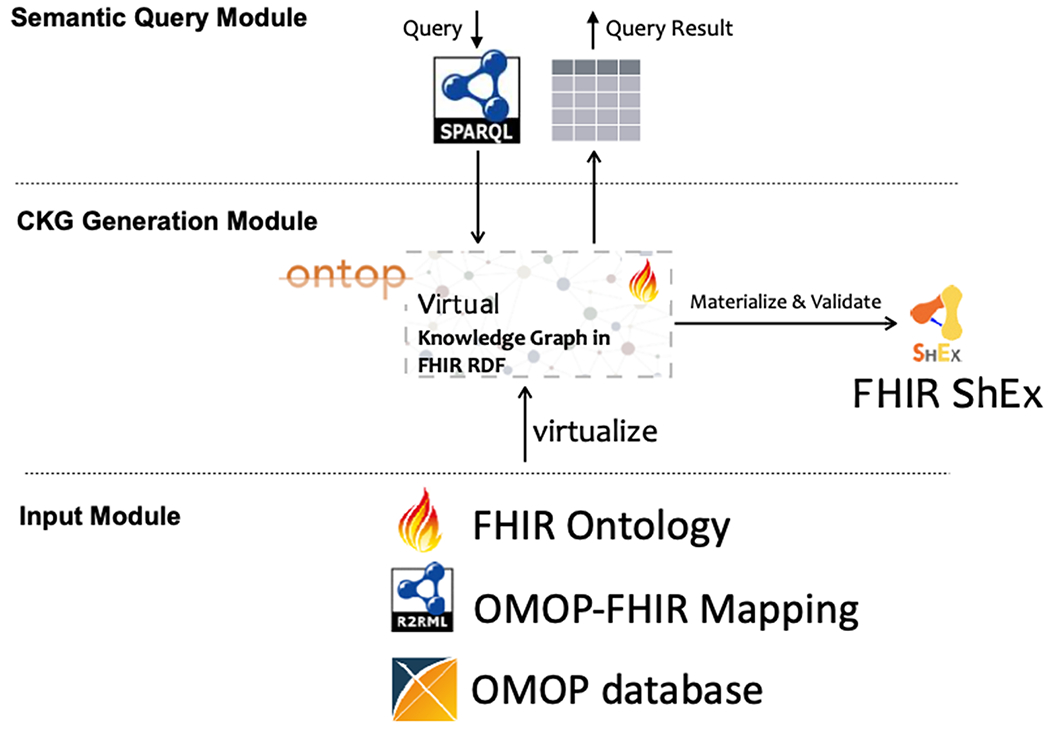
System architecture of the FHIR-Ontop-OMOP system.

We developed a system called FHIR-Ontop-OMOP to generate virtual clinical KGs against the OMOP CDM relational databases. Fig. 3 shows the system architecture of the FHIR-Ontop-OMOP system. The system consists of the following modules (from the bottom to up): 1) an input module that takes input from the FHIR model ontology, the OMOP data repository, and OMOP-FHIR mappings represented by a mapping template; 2) a CKG generation module that relies on the Ontop system to generate a virtual CKG; and 3) a semantic query module that establishes SPARQL endpoints with reasoning capability.

### 3.2. Input module

At the bottom in Fig. 3, it is the *OMOP relational database* to be mapped to RDF. FHIR-Ontop-OMOP system can be implemented seamlessly against any OMOP database, making the system portable. *The FHIR Model Ontology* serves as a catalog of standard URIs for all FHIR model artifacts. This ontology defines a high-level global schema of clinical data sources and provides a standard vocabulary for user queries.

*The most complex component is the OMOP-FHIR mapping*, which specifies the correspondence between the data models of the relational data sources in OMOP CDM and the RDF graph in FHIR RDF. In this study, we are focused on the mappings between the OMOP CDM and the FHIR RDF graph. Ontop supports the R2RML standard mapping language and the Ontop mapping language which is fully interoperable with R2RML [31].

**Fig. 4.**
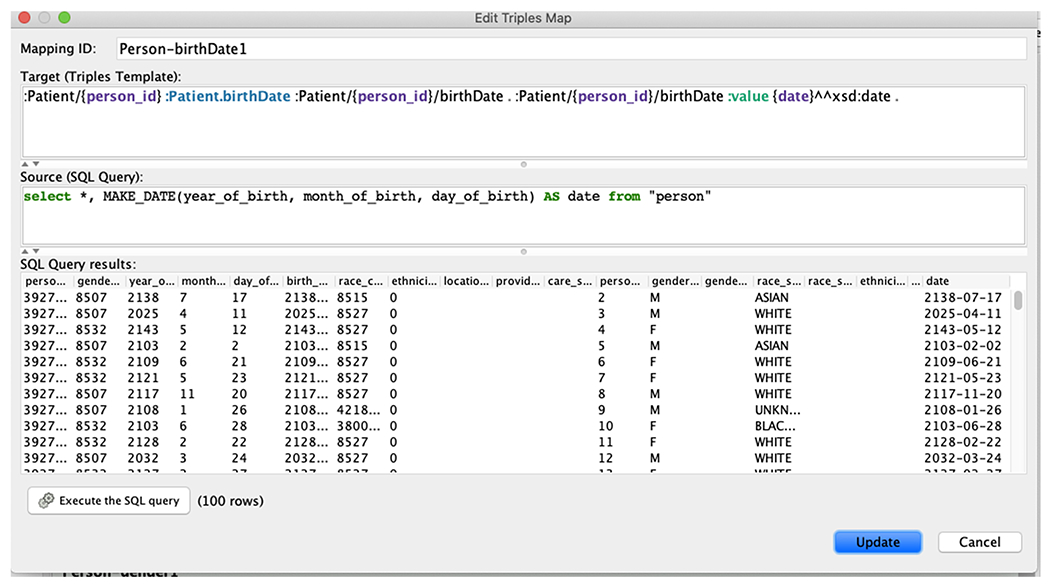
A Protege screenshot illustrating the creation of mappings between three fields of the OMOP person table and the FHIR Patient birthDate.

At the early stage of the system implementation, we used the Protege plugin Ontop Mappings to manually create an initial set of mappings to test the feasibility of the FHIR-Ontop-OMOP system. The typical process includes the following steps: 1) establishing a database connection with an OMOP database by setting up the connection parameters using the Connection Parameters panel; 2) selecting the properties to be used in defining mappings using the Ontop Properties panel; 3) creating mappings using the Mapping Manager panel (Fig. 4), in which SQL queries can be executed against the OMOP database to help understand the patient data.

At a later stage of the system implementation, we realized that the mapping creation process is more efficient if we can (semi)-automate the mapping generation, especially for the FHIR standard that extensively uses intermediate nodes. For example, it is tedious to write nodes like: Patient/{person_id}/birthDate in the example in Fig. 4.

We developed a two-step approach for the automation. We created a user-friendly mapping template in the RDF Turtle format, which we call it a Turtle Template Mapping Language (TML), to encode the data model mappings between the OMOP CDM and FHIR RDF. We also implemented a Java-based converter that translates the mappings defined in TML into the Ontop mappings that are required for the Ontop system.

**Fig. 5.**
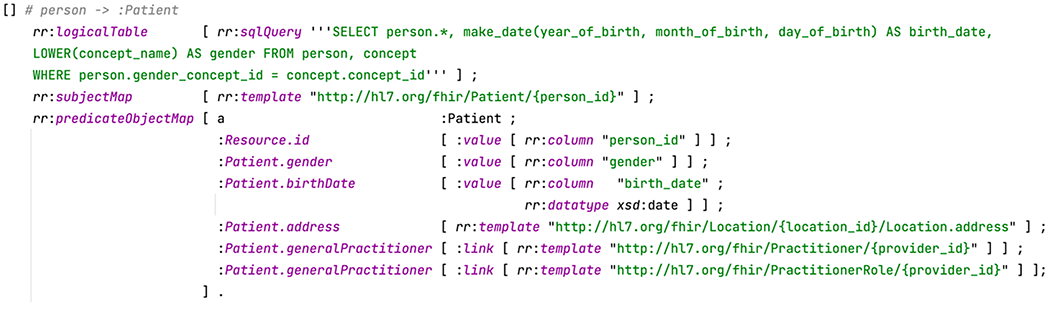
An example TML mapping entry defined between the OMOP Person table and the FHIR Patient Resource using the Turtle mapping template.

We show how the TML mapping works through an example mapping entry in Fig. 5 between the OMOP Person table and the FHIR Patient Resource. The first two components rr:logicalTable and rr:subjectMap behave identical to R2RML: rr:logicalTable specifies a data source (a SQL query involving the person table) to be mapped to RDF, and rr:subjectMap specifies an IRI template for the subject (a string with the placeholder {person_id}, where person_id is a column in the SQL query). The last part rr:predicateObjectMap (diverged from R2RML) shows a list of predicates and objects. It defines the field level mappings and its structure that follows directly to the FHIR ShEx schema.

In order to develop OMOP-FHIR mapping, we reviewed available mappings created by a number of research groups including 1) the OHDSI FHIR Workgroup; 2) the Common Data Model Harmonization Project; 3) the Georgia Tech‘s OMOP-on-FHIR project; and 4) the FHIR DAF Research Implementation Guide team [32–35]. We harvested the set of mappings that have a consensus across these groups and used them to populate the TML mapping. In addition, to represent Concept information from OMOP CDM, we used the FHIR CodeableConcept data type for this purpose. The FHIR specification defines a set of data types that are used for the resource elements. CodeableConcept is a complex data type used to represent a value that is usually supplied by providing a reference to one or more terminologies or ontologies but may also be defined by the provision of text. Mapping CodeableConcept to the Concept information from OMOP CDM provides a natural way to link health data with standard concept annotations. For example, Condition. code is restricted by the data type CodeableConcept. We can assign a coded value sct:39065001 from SNOMED CT or the provision of text “Burnt Ear” using CodeableConcept to describe a condition instance.

### 3.3. CKG generation module

**Fig. 6.**
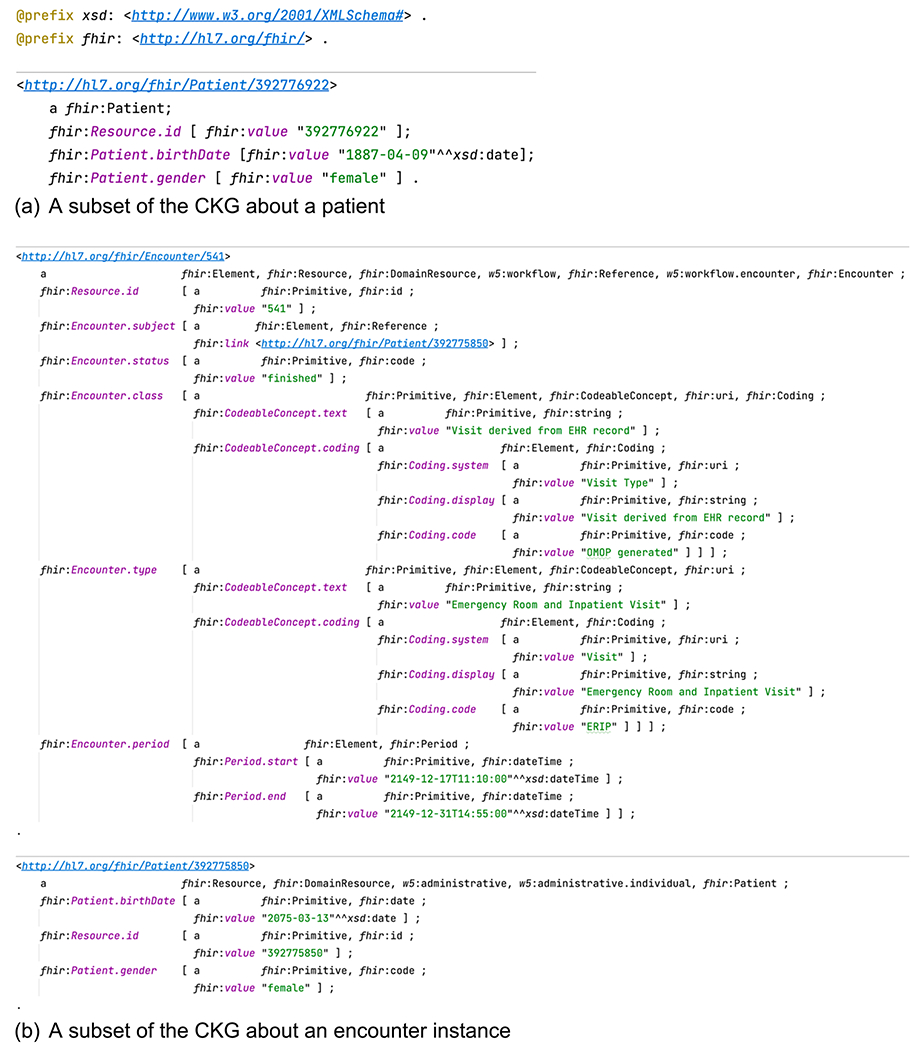
Examples of clinical knowledge graphs generated.

This module uses the Ontop system to generate a virtual CKG over the input module. Fig. 6 (a) shows an example illustrating the converted CKG in FHIR RDF from an answer to the SQL query in Fig. 5. Note that this CKG does not contain information about address and practitioner as the corresponding columns in the databases contain only NULL values. Fig. 6 (b) shows a more complex example about the CKG of an Encounter instance.

The CKG conformance to the FHIR RDF specification is realized through the Ontop mappings as defined in the Turtle mapping template (Fig. 5). This conformance is also validated using the FHIR RDF validation tool known as ShEx validator developed in our previous studies [15].

The virtual CKGs do not require additional storage space. A virtual CKG just wraps an existing relational database as a virtual CKG. This virtual CKG is only accessible at query answering time. This is advantageous because a classical materialization-based approach is very costly in terms of both materialization time and disk space.

### 3.4. Semantic query module

**Fig. 7.**
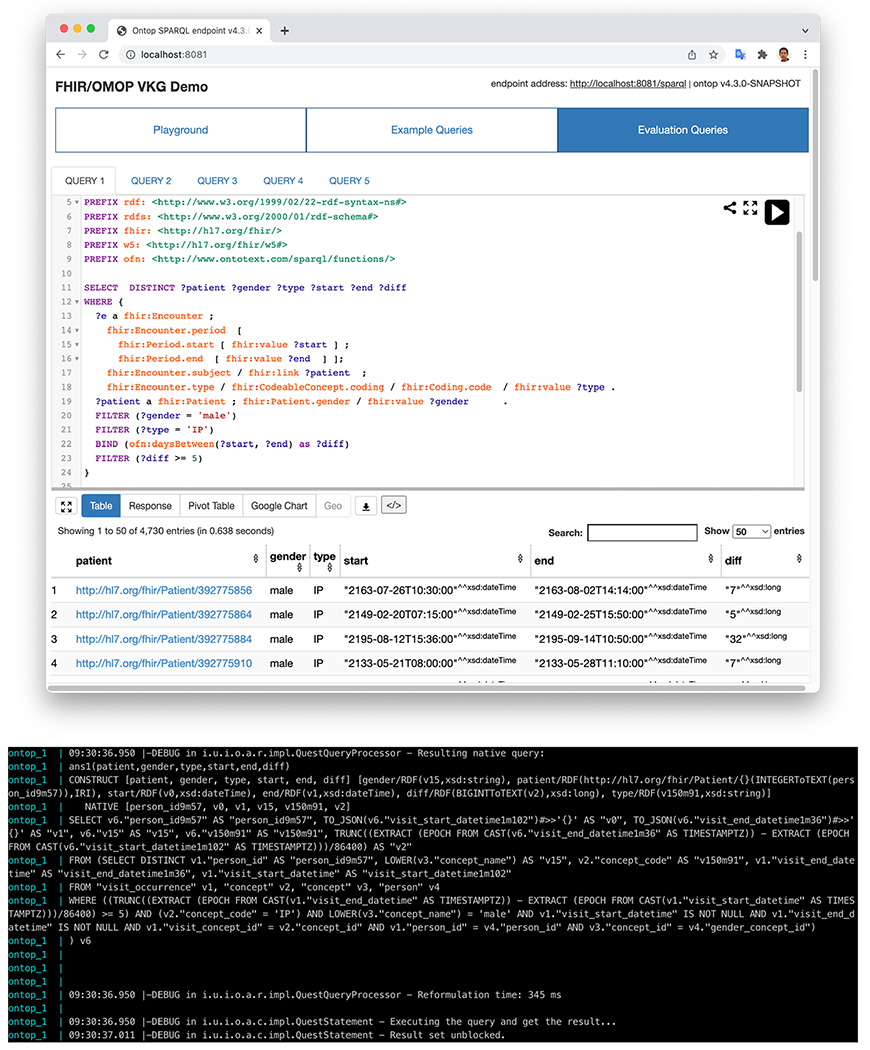
A SPARQL query example against the virtual CKG converted from the MIMIC III OMOP database, and part of the log information containing the SQL translation.

This module relies on the query answering interface of Ontop. The Ontop system translates SPARQL queries over the CKG to SQL queries over the OMOP database, using the FHIR ontology and FHIR-OMOP mapping. Fig. 7 shows a SPARQL query example (Query 1 in the Evaluation section) against the MIMIC III OMOP database, and its corresponding SQL translation.

### 3.5. Evaluation design

We evaluated the system in terms of the faithfulness of data transformation, and the conformance of the generated CKGs to the FHIR RDF specification. We implemented the system against the MIMIC-III OMOP CDM data and used the generated CKG in FHIR RDF for the evaluation.

### 3.6. Faithfulness of data transformation

We first tested the faithfulness of data transformation from the OMOP CDM to the CKGs in FHIR RDF. We manually wrote five demonstration SQL queries designed to make use of a variety of tables, columns, and data types across the OMOP model, shown in Table 1. We then followed the FHIR specification to write equivalent SPARQL queries using the same logic as the SQL queries, for the purpose of comparing the output. Faithful transformation entails that the patients identified by the SQL and SPARQL queries be identical.

### 3.7. Conformance of the CKGs to the FHIR RDF ShEx specification

We tested the conformance of the generated CKGs to the FHIR RDF ShEx specification. We used the shex-validate command line utility from the shexjs library, which is a validation tool developed in the previous studies [36]. We generated a subset of the CKG for 100 patients out of the system implemented for the MIMIC-III dataset and materialized them in RDF triples using Ontop. To do so, we reused the same mapping for query answering but adding the appropriate filters over person IDs on the SQL queries to choose the patients. This data set includes the instances of 100 Patients, 1,457 Conditions, 1,855 Procedures, 74,598 MedicationStatements, 808,198 Observations, and 2,069 relevant CodeableConcepts. This sub-dataset has been materialized by Ontop. The generated turtle format of these files took up 4.1 GB of disk space and were then loaded into the shexjs library for validation. We tested the conformance of the sub-CKG to the FHIR RDF specifications of a number of clinical FHIR resources including Patient, Condition, Procedure, MedicationStatement, Observation, and CodeableConcept.

## 4. Results

### 4.1. System implementation status

A beta version of the FHIR-Ontop-OMOP system has been released at the project GitHub site at https://github.com/fhircat/FHIROntopOMOP, which includes a docker-based installation. We implemented the system against the MIMIC-III OMOP CDM data and exposed it as a queryable CKG compliant with the HL7 FHIR standard using the Ontop. The virtual CKG in FHIR RDF contains triples describing 46,520 instances of FHIR Patient, 716,595 instances of Condition, 1,063,525 instances of Procedure, 24,934,751 instances of MedicationStatement, 365,181,104 instances of Observations, and 4,779,672 instances of CodeableConcept, among others.

### 4.2. Mappings implemented

Table 2 shows high-level mappings between OMOP tables and FHIR resources implemented in the system. The detailed element mappings are available in the supplemental tables Table S1 and Table S2. These mappings consist of a total of more than 100 data elements from 5 clinical data tables (Person, Condition_occurrence, Drug_exposure, Procedure_occurrence, and Measurement), 3 health system tables (Visit_occurrence, Location and Provider) and 3 vocabulary tables (Concept, Concept_relationship and Concept_ancestor). The mappings covered data elements from 11 FHIR resources (Patient, Encounter, Location, Condition, MedicationStatement, Observation, Procedure, Practitioner, CodeableConcept, Coding, ConceptMap). We note that each OMOP data element is normally mapped to one FHIR data element (e.g., person_id to Resource.id), but sometimes also generates intermediate blank nodes (e.g. visit_start_datetime to Encounter.period / Period.start). We also observe that the OMOP tables often contain some redundancy, e.g., (1) in addition to birth_datetime, the Person table also stores year, month and day of birth in separate columns, and (2) time related information is given in two columns (e.g., visit_start_date and visit_start_datetime) with different precisions. We do not need to map these redundant columns and we mark the implementation status as “not applicable”.

### 4.3. Evaluation results

#### 4.3.1. Faithfulness of data transformation

Table 3 shows the results of running our demonstration queries against MIMIC III data using SQL directly against OMOP and SPARQL via the FHIR-Ontop-OMOP system. The counts for all queries are identical, ensuring faithful transformation of the tested domains.

#### 4.3.2. Conformance of the CKGs to the FHIR RDF specification

The evaluation result showed that the generated RDF triples for 100 patients were fully compliant with the FHIR RDF ShEx specification. This result was not surprising because the turtle-template mapping follows directly to the structure of the ShEx specification, which guarantees the conformance.

## 5. Discussion

Achieving interoperability between FHIR and OMOP has been desired by the standardization and research communities. Notably, HL7 and OHDSI recently announced a collaboration to address the sharing and tracking of data in the healthcare and research industries by creating a single standard data model [37]. The development of the Semantic Web-based FHIR-Ontop-OMOP system in this study provides a meaningful use case for such a collaboration, demonstrating great potential in healthcare AI applications enabled by the interoperability of FHIR and OMOP CDM.

Mapping creation is a non-trivial time-consuming task. In this study, we intended to create a new mapping language that can represent the data model mappings in a manner that is both human friendly and machine processable. We reused existing features from the standard W3C R2RML language and the Ontop mapping language [31]. We argued that these two languages do not completely meet our needs. Being designed as a machine exchange format, R2RML is very verbose, and difficult to read and write by humans. The Ontop mapping language is already much more compact and readable (as shown in Fig. 4), but it is not able to directly express the nested structure commonly used in FHIR. This becomes even more complex when dealing with multiple levels of nesting. Seeing the limitations of existing languages, we created a new template language: turtle-template mapping language (TML). Syntactically, a TML mapping is written as a Turtle document, and consists of multiple TML entries. Intuitively, if we view each mapping as a tree, the root level constructs (rr:logicalTable, rr:subjectMap, and predicateObjectMap) and the leaf level constructs (rr:column, rr:termType, rr:datatype, rr:template) work exactly the same with R2RML. However, we have changed the middle of this tree to the turtle template (which is closer to the style of Ontop mapping). In this implementation, its structure is identical to the template used in the FHIR specification.

Both faithfulness and conformance evaluations were an iterative procedure. For the faithfulness evaluation, we first noticed that formulating SQL and SPARQL queries use slightly different methodologies. The SQL version tends to use the hard-coded “magic” value directly, e.g., p.gender_concept_id = 8507 for selecting male patients in Q1, while the SPARQL version uses the FHIR gender identity in (?gender = ’male’). This makes the SPARQL version more readable. Some SPARQL queries were initially difficult to formulate or did not show identical counts to the SQL queries. By looking into the underlying reasons, we were able to identify and fix a number of implementation issues of FHIR-Ontop-OMOP and the Ontop engine. Notably, Q1 requires datetime functions to compute the duration between two datetime values. However, this is not part of the standard SPARQL functions, and was not supported by Ontop. To address this, we have implemented such functions in Ontop following the corresponding functions of GraphDB [38]. Also for Q1, when identifying inpatient admissions, according to FHIR, the code for inpatient status should be “IMP”, but the actual value in the MIMIC-OMOP database is “IP”. Therefore we have to use the filter?type=“IP” as a workaround. For Q5 (Patients with an HbA1c >= 10 %), there is a subtle difference between using date and datetime values. The SQL version could use the measurement_date column directly. Instead, in FHIR-Ontop-OMOP, the mapping uses the column measurement_datetime for all the observations. This query needs to deal with the case that one person within one day has two different measurements of the same value. Therefore, we need to extract the date information from the column measurement_datetime. For the conformance evaluation, we identified some minor issues in the current R4 version specification of the FHIR RDF ShEx standard. Specifically, a number of datatype shapes (e.g., <https://hl7.org/fhirpath/System.String>) are underspecified and there are also inconsistent behaviors in these datatypes regarding whether a “fhir:value” intermediate edge is needed. We manually fixed them by adding a few “catch all” ShEx shapes: <https://hl7.org/fhirpath/System.String>. These issues have been reported to the maintainers of the FHIR RDF ShEX specification and are going to be fixed in the next major release R5.

This study is limited by several factors. First, we harvested and implemented a preliminary consensus set of mappings between FHIR and OMOP CDM created by different groups, which covers main clinical FHIR resources (e.g., Condition, Procedure, MedicationStatement, Observation). We understand that the collaboration between HL7 and OHDSI in the future may produce a set of mappings that are more robust and can be used to enhance the current system. Second, we used the FHIR resource CodeableConcept to represent standard concepts captured in the OMOP CDM Concept table (see Table S2), in which vocabulary_id is mapped to fhir:Coding.system and concept_code is mapped to fhir:Coding.code. This strategy worked well on most scenarios where standard vocabularies such as SNOMED CT, ICD10CM, RxNorm, or CPT were used. However, we should note that in the current system we have not implemented a translation from the OMOP vocabulary_id into preferred Coding System used in FHIR (https://www.hl7.org/fhir/terminologies-systems.html). For example, in the OMOP vocabulary_id for SNOMED CT is “SNOMED”, which is directly used in FHIR Coding.system. However, a preferred Coding System URI is https://snomed.info/sct. This can be fixed by defining a mapping table to associate the preferred Coding System URIs of externally published Coding Systems in FHIR with the vocabulary_id from OMOP. In addition, for the domains like Encounter, the definitions of encounter type between HL7 FHIR and OMOP CDM are different. For example, the code “IMP” is defined in a HL7 v3 valueset for inpatient encounter, whereas two concept_ids (ie, 9201, and 262) are used in the MIMIC-III OMOP CDM to define inpatient visit. This means that the mappings in the valueset level need to be handled in the system. In the future study, we plan to systematically analyze mappings needed in the valueset level and implement such mappings in the system. To understand the degree of interoperability between OMOP CDM and FHIR, we identified code systems (https://www.hl7.org/fhir/terminologies-systems.html) and valuesets (https://www.hl7.org/fhir/terminologies-valuesets.html) from the following links in the current FHIR specification. A total of 45 externally published code systems, 293 internal code systems, and 721 valuesets were identified. Out of 45 externally published code systems, 13 (29 %) can be mapped to the OMOP vocabulary ids, consisting of SNOMED, RxNorm, LOINC, UCUM, CPT4, NDFRT, NDC, CVX, ICD[x], ATC, NUCC, HGNC and ClinVar. This means about 70 % of preferred code systems in FHIR did not have their corresponding vocabularies in OMOP CDM. For those unmapped OMOP vocabulary ids, we can use the existing OMOP convention for now to represent them in Coding.system. None of 293 internal code systems and 721 valuesets has been mapped to the OMOP vocabularies. Further community-based harmonization may still be needed for these internal code systems and valuesets. Third, we only used one single MIMIC-III OMOP CDM instance for the evaluation. As the next step, we plan to identify multiple clinical data repositories in OMOP CDM for more rigorous evaluation, including demonstrating distributed analytics and AI applications enabled by the system.

## 6. Conclusion

The FHIR-Ontop-OMOP system provides a meaningful use case demonstrating the potential that can be enabled by the interoperability between FHIR and OMOP CDM. Generated clinical knowledge graphs in FHIR RDF provide a semantic foundation to enable explainable AI applications in healthcare.

In the future, we plan to leverage FHIR-Ontop-OMOP to build FHIR data services and applications. For example, simple RESTful APIs over RDF graphs can be established to support a large community of web developers by using the Linked Data APIs [39]. The Linked Data APIs enable representing resources in simple RDF, JSON, XML, and CSV formats with various selection criteria. We also plan to demonstrate advanced features of the system (e.g., inference capability, federated semantic queries, distributed analytics, and AI applications) empowered by FHIR, clinical knowledge graphs, and the Semantic Web technologies. Finally, we want to study the Semantic Web and explainable AI applications as we described in the Introduction section. For example, federated clinical knowledge graph embeddings can be potentially realized using CKGs generated from the FHIR-Ontop-OMOP system across multiple OMOP CDM instances.

## Supplementary Material

supplemental table

## Figures and Tables

**Table 1:** Demonstration Queries.

OMOP Table(s)	FHIR Resource(s)	Query*
person, visit_occurrence, concept	Patient, Encounter	Q1: Identify male patients with inpatient admissions lasting greater than 5 days.
condition_occurrence, concept	Condition	Q2: Identify patients diagnosed with Alzheimer’s Disease.
procedure_occurrence, concept	Procedure	Q3: Identify patients who delivered a baby.
drug_exposure, concept	Medication Statement	Q4: Identify patients prescribed trazadone.
measurement, concept	Observation	Q5: Identify patients with an HbA1c result >= 10 %.

* SQL and SPARQL code versions of these queries can be reviewed at https://github.com/fhircat/FHIROntopOMOP/blob/main/evaluation/jbi-2022-queries.md.

**Table 2:** High-level mappings between OMOP tables and FHIR resources. Note that detailed element mappings are available in the supplemental tables Table S1 and Table S2.

OMOP Table	FHIR Resource
PERSON	Patient
VISIT_OCCURENCE	Encounter
CARE_SITE	Location
CONDITION_OCCURENCE	Condition
DRUG_EXPOSURE	MedicationStatement
LOCATION	Location
MEASUREMENT	Observation
PROCEDURE_OCCURENCE	Procedure
PROVIDER	Practitioner/PractitionerRole
CONCEPT	CodeableConcept/Coding
CONCEPT_RELATIONSHIP	ConceptMap
CONCEPT_ANCESTER	ConceptMap

**Table 3:** Demonstration query results.

Query	SQL patient count	SPARQL patient count
Q1: Identify male patients with inpatient admissions lasting greater than 5 days.	4730	4730
Q2: Identify patients diagnosed with Alzheimer’s Disease.	569	569
Q3: Identify patients who delivered a baby.	34	34
Q4: Identify patients prescribed trazadone.	6737	6737
Q5: Identify patients with an HbA1c result ≥ 10 %.	944	944
