# Supplementary material for: FHIR-Ontop-OMOP: Building clinical knowledge graphs in FHIR RDF with the OMOP Common data Model
Source: J Biomed Inform. Author manuscript; Available in PMC 2023 Oct 1. (PMC9561043; doi:10.1016/j.jbi.2022.104201)
Supplement: supplemental table [file NIHMS1839711-supplement-supplemental_table.docx]

Table S1. Mappings implemented for the OMOP CDM clinical data and health system tables

| **OMOP Table/Column** | **FHIR Resource/Element** | **Implementation Status** |
| --- | --- | --- |
| Table: Person -> FHIR Resource: Patient | |  |
| person_id | Resource.id | Yes |
| provider_id | Patient.generalPractitioner | Yes |
| care_site_id | Patient.managingOrganization | Yes |
| gender_concept_id | Patient.gender | Yes |
| year_of_birth | Patient.birthDate | Yes |
| month_of_birth | Patient.birthDate | Yes |
| day_of_birth | Patient.birthDate | Yes |
| birth_datetime |  | Not applicable |
| race_concept_id | Patient.extension: us-core-race | To be implemented |
| ethnicity_concept_id | Patient.extension: us-core-ethnicity | To be implemented |
| location_id | Patient.address | Yes |
| Table: VISIT_OCCURRENCE -> FHIR Resource: Encounter | |  |
| visit_occurence_id | Resource.id |  |
| preceding_visit_occurence | Encounter.partOf | Yes |
| person_id | Encounter.subject | Yes |
| visit_concept_id | Encounter.type | Yes |
| visit_start_date |  | Not applicable |
| visit_start_datetime | Encounter.period/Period.start | Yes |
| visit_end_date |  | Not applicable |
| visit_end_datetime | Encounter.period/Period.end | Yes |
| visit_type_concept_id | Encounter.class | Yes |
| provider_id | Encounter.performer | Yes |
| Table: CARE_SITE -> FHIR Resource: Location | |  |
| care_site_id | Resource.id | Yes |
| care_site_name | Location.name | Yes |
| place_of_service_concept_id | Location.type | Yes |
| location_id | Location.address | Yes |
| Table: CONDITION_OCCURRENCE -> FHIR Resource: Condition | |  |
| condition_occurrence_id | Resource.id | Yes |
| provider_id | Condition.asserter | Yes |
| visit_occurrence_id | Condition.encounter | Yes |
| condition_status_concept_id | Condition.clinicalStatus | Yes |
| person_id | Condition.subject | Yes |
| condition_concept_id | Condition.code | Yes |
| condition_start_date |  | Not applicable |
| condition_start_datetime | Condition.onsetDateTime | Yes |
| condition_end_date |  | Not applicable |
| condition_end_datetime | Condition.abatementDateTime | Yes |
| condition_type_concept_id | Condition.category | Yes |
| Table: DRUG_EXPOSURE -> FHIR Resource: MedicationStatement | |  |
| drug_exposure_id | Resource.id | Yes |
| stop_reason | MedicationStatement.statusReason | Yes |
| visit_occurrence_id | MedicationStatement.context | Yes |
| person_id | MedicationStatement.subject | Yes |
| drug_concept_id | MedicationStatement.medicationCodeableConcept | Yes |
| drug_exposure_start_date |  | Not applicable |
| drug_exposure_start_datetime | MedicationStatement.effectivePeriod/Period.start | Yes |
| drug_exposure_end_date |  | Not applicable |
| drug_exposure_end_datetime | MedicationStatement.effectivePeriod/Period.end | Yes |
| drug_type_concept_id | MedicationStatement.catagory | Yes |
| Table: LOCATION -> FHIR Resource: Location | |  |
| location_id | Resource.id | Yes |
| address_1 | Location.address.line | Yes |
| address_2 | Location.address.line | Yes |
| city | Location.address.city | Yes |
| state | Location.address.state | Yes |
| zip | Location.address.postalCode | Yes |
| country | Location.address.country | Yes |
| Table: MEASUREMENT -> FHIR Resource: Observation | |  |
| measurement_id | Resource.id | Yes |
| unit_concept_id | Observation.valueQuantity/Quantity.unit | Yes |
| range_low | Observation.referenceRange/ Observation.referenceRange.low | Yes |
| range_high | Observation.referenceRange/ Observation.referenceRange.high | Yes |
| provider_id | Observation.performer (Practitioner) | Yes |
| visit_occurrence_id | Observation.encounter | Yes |
| person_id | Observation.subject | Yes |
| measurement_concept_id | Observation.code | Yes |
| measurement_date |  | Not applicable |
| measurement_datetime | Observation.effectiveDateTime | Yes |
| measurement_type_concept_id | Observation.category | Yes |
| value_as_number | Observation.valueQuantity/Quantity.unit | Yes |
| value_as_concept_id | Observation.valueCodeableConcept | Yes |
| Table: PROCEDURE_OCCURRENCE -> FHIR Resource: Procedure | |  |
| procedure_occurrence_id | Resource.id | Yes |
| visit_occurrence_id | Procedure.encounter | Yes |
| person_id | Procedure.subject | Yes |
| procedure_concept_id | Procedure.code | Yes |
| procedure_date |  | Not applicable |
| procedure_datetime | Procedure.performedDateTime | Yes |
| procedure_type_concept_id | Procedure.category | Yes |
| provider_id | Procedure.performer.actor | Yes |
| Table: PROVIDER -> FHIR Resource: Practitioner/PractitionerRole | |  |
| provider_id | Resource.id | Yes |
| provider_name | Practitioner.name | Yes |
| npi | Practitioner.identifier | Yes |
| dea | Practitioner.qualification | Yes |
| specialty_concept_id | PractitionerRole.specialty | Yes |
| care_site_id | PractitionerRole.location | Yes |
| provider_id | PractitionerRole.practitioner | Yes |
| year_of_birth | Practitioner.birthDate | Yes |
| gender_concept_id | Practitioner.gender | Yes |

Table S2. Mappings implemented for the OMOP vocabulary tables

| **OMOP Table/Column** | **FHIR Resource/Element** | **Implementation Status** |
| --- | --- | --- |
| Table: CONCEPT-> FHIR Element: Coding/CodeableConcept | |  |
| concept_id | CodeableConcept | Yes |
| concept_code | CodeableConcept.coding/Coding.code | Yes |
| concept_name | CodeableConcept.coding/Coding.display | Yes |
| vocabulary_id | CodeableConcept.coding/Coding.system | Yes |
| concept_name | CodeableConcept.text | Yes |
| Table: CONCEPT_RELATIONSHIP -> FHIR Resource: ConceptMap | |  |
| concept_id_1 | Resource.id | Yes |
| concept_id_2 | Resource.id | Yes |
| concept_id_1 | ConceptMap.sourceUri | Not applicable |
| concept_id_2 | ConceptMap.targetUri | Not applicable |
| concept_id_1 | ConceptMap.group.source | Yes |
| concept_id_2 | ConceptMap.group.target | Yes |
| relationship_id | ConceptMap.group.element.target.equivalence | Yes |
| Table: CONCEPT_ANCESTER -> FHIR Resource: ConceptMap | |  |
| ancestor_concept_id | Resource.id | Yes |
| descendant_concept_id | Resource.id | Yes |
| ancestor_concept_id | ConceptMap.sourceUri | Not applicable |
| descendant_concept_id | ConceptMap.targetUri | Not applicable |
| ancestor_concept_id | ConceptMap.group.source | Yes |
| descendant_concept_id | ConceptMap.group.target | Yes |
| "subsumes" | ConceptMap.group.element.target.equivalence | Yes |
